# Reply: non-significant results do not imply similarity or safety in NC-FET follicle-size comparisons: a clarification

**DOI:** 10.1093/hropen/hoag008

**Published:** 2026-01-30

**Authors:** Jie Zhang, Ling Wu

**Affiliations:** Department of Assisted Reproduction, Shanghai Ninth People’s Hospital, Shanghai Jiao Tong University School of Medicine, Shanghai, China; Department of Assisted Reproduction, Shanghai Ninth People’s Hospital, Shanghai Jiao Tong University School of Medicine, Shanghai, China

Dear Editor,

We would like to thank Dr Iga for his thoughtful and constructive comments (Iga, 2026) on our article ‘Pregnancy and perinatal outcomes after modified natural cycle-frozen embryo transfers according to size of the dominant follicle on the hCG trigger day’. (Zhang *et al*, 2025). We appreciate his interest in our work and the opportunity to clarify several aspects of our study.

First, our study (Zhang *et al*, 2025) employed a retrospective design rather than a non-inferiority trial. Accordingly, our conclusions focused on the absence of a statistically significant difference. Although non-significance does not prove equivalence, pregnancy rates were similar, and adjusted analyses showed that no significant differences were observed in any of the pregnancy outcomes when comparing the reference group to the other follicle size categories. Moreover, when treating follicle size as a continuous variable, there remained no significant association between the follicle size and any reproductive outcomes. Additionally, several sensitivity analyses have been conducted, and the results are consistent with the main findings.

Second, we acknowledge that the sample sizes in certain subgroups, particularly for the <12 mm group, were small, which may limit the statistical power to identify differences among some groups. This limitation has been discussed in our article (Zhang *et al*, 2025), and we further highlighted that caution should be taken when interpreting these findings. Further prospective research with larger sample sizes is necessary to confirm our findings.

Finally, we fully agree that Wilson intervals for single proportions and Newcombe’s method for between-group differences are preferable to traditional Wald intervals; however, with small sample sizes, these methods still yield wide intervals that reflect uncertainty. Consequently, we call for future larger studies employing a broader range of statistical approaches to examine pregnancy outcomes associated with follicle size.

We thank Dr Iga again for the valuable comment and appreciate this opportunity to engage in this important dialogue.

## References

[hoag008-B1] Iga K. Non-significant results do not imply similarity or safety in NC-FET follicle-size comparisons: a clarification. Hum Reprod Open 2026;2026:hoag007. 41696678 10.1093/hropen/hoag007 PMC12906230

[hoag008-B2] Zhang J, Qiu S, Gao H, Mao X, Guo Y, Wu L. Pregnancy and perinatal outcomes after modified natural cycle-frozen embryo transfers according to size of the dominant follicle on the hCG trigger day. Hum Reprod Open 2025;2025:hoaf047. 40799621 10.1093/hropen/hoaf047 PMC12343029

